# China’s forests host a vast, previously unquantified population of old trees

**DOI:** 10.1093/nsr/nwag197

**Published:** 2026-04-11

**Authors:** Yu Ren, Kai Cheng, Junmin Zhang, Tianyu Xiang, Yuting Xu, Shu Yang, Ying Li, Ouya Fang, Qiuli Yang, Ang Chen, Yu Li, Zhixin Cheng, Heng Zhang, Guangcai Xu, Yongchuan Yang, Qinghua Guo

**Affiliations:** Institute of Remote Sensing and Geographic Information System, School of Earth and Space Sciences, Peking University, China; Institute of Remote Sensing and Geographic Information System, School of Earth and Space Sciences, Peking University, China; College of Environment and Civil Engineering, Chengdu University of Technology, China; College of Geography and Planning, Chengdu University of Technology, China; Lin’an Branch of Hangzhou Ecology and Environment Bureau, China; Lin’an Branch of Hangzhou Ecology and Environment Bureau, China; Beijing Institute of Landscape Architecture and Traditional Architectural Design and Research Co. Ltd., China; Key Laboratory of Vegetation and Environmental Change, Institute of Botany, Chinese Academy of Sciences, China; College of Geography and Remote Sensing Science, Xinjiang University, China; Xinjiang Key Laboratory of Oasis Ecology, Xinjiang University, China; Xinjiang Field Scientific Observation and Research Station for the Oasisization Process in the Hinterland of the Taklamakan Desert, China; Institute of Remote Sensing and Geographic Information System, School of Earth and Space Sciences, Peking University, China; Institute of Remote Sensing and Geographic Information System, School of Earth and Space Sciences, Peking University, China; Institute of Remote Sensing and Geographic Information System, School of Earth and Space Sciences, Peking University, China; Beijing GreenValley Technology Co., Ltd, China; Beijing GreenValley Technology Co., Ltd, China; Key Laboratory of the Three Gorges Reservoir Region’s Eco-Environment, Ministry of Education, Chongqing University, China; Institute of Remote Sensing and Geographic Information System, School of Earth and Space Sciences, Peking University, China

Old trees not only preserve profound historical and ecological records but also play a critical role in sustaining ecosystem functions [1]. They provide distinct microenvironments for a wealth of biodiversity, while simultaneously storing massive carbon stocks and regulating hydrological cycles [2]. Amidst accelerating climate change and widespread habitat loss, old trees face mounting threats. Effective conservation requires a fundamental understanding of their distributions; however, the spatial patterns of old trees remain poorly quantified, posing a significant obstacle to their protection.

China harbours one of the world’s richest reservoirs of old trees. To date, two national surveys in China have been conducted, with the second documenting 5.08 million ancient and famous trees, primarily in human-dominated landscapes (in urban and rural areas) [3]. Local traditions and cultural beliefs have been crucial for preserving at least 1580 species of old trees in China, despite the significant challenges to their long-term survival in populated areas [4,5]. However, vast tracts of forests—where the old trees often persist—remain largely unquantified. The ongoing third national survey in China broadens its scope to explicitly include old-tree groups within vast forest ecosystems. Crucially, the foundational and spatially explicit data needed for this mission remain elusive. To address this critical knowledge gap, we develop the first high-resolution (1-hectare) map of old trees across China’s forests to answer the fundamental questions: How many old trees are in China’s forests, and where are they?

We first established the key definitions for our analysis: old trees (trees over 100 years old) and old-tree groups (requiring a minimum density of 20 old trees/ha). These definitions were adopted to align precisely with the official Chinese national standard being implemented in the ongoing third national survey of ancient and famous tree resources, thereby providing a robust baseline. Consistent with these standards, our analysis focuses on forest ecosystems, excluding the human-dominated landscapes covered by previous censuses. We estimated the quantity of old trees at the pixel level by modeling the forest age structure using the two-parameter Weibull distribution (detailed in the supplementary material). To retrieve the distribution parameters for each pixel and estimate the number of old trees, we developed a unified probabilistic inference framework based on structural moment inversion. This framework integrates multi-source nationwide remote sensing datasets, including tree density, forest age, and key structural

metrics—specifically, arithmetic and weighted mean tree heights (Table S1)—all unified to 100 m resolution. A key feature of our method is the characterization of age structure heterogeneity within a pixel using a structural heterogeneity index, defined as the ratio of the weighted mean to the arithmetic mean heights. By leveraging Monte Carlo simulations, we developed nonlinear inversion models to estimate the shape and scale parameters of the Weibull distribution directly from structural moments, enabling us to calculate the specific survival probability of old trees and generate a seamless 100 m national map of old tree density (Fig. 1a), from which the distribution map of old-tree groups was subsequently delineated (Fig. S1). Map accuracy was validated from two aspects: accuracy for old-tree presence and identification accuracy for old-tree groups, using two ground-truth datasets (Table S2). The results indicated overall accuracies of 89.97% for classifying old trees and 81.69% for identifying old-tree groups. While confirming spatial patterns, pixel-level densities should be interpreted as probabilistic estimates rather than precise counts, given the lack of exhaustive ground-truth data.

**Figure 1.**
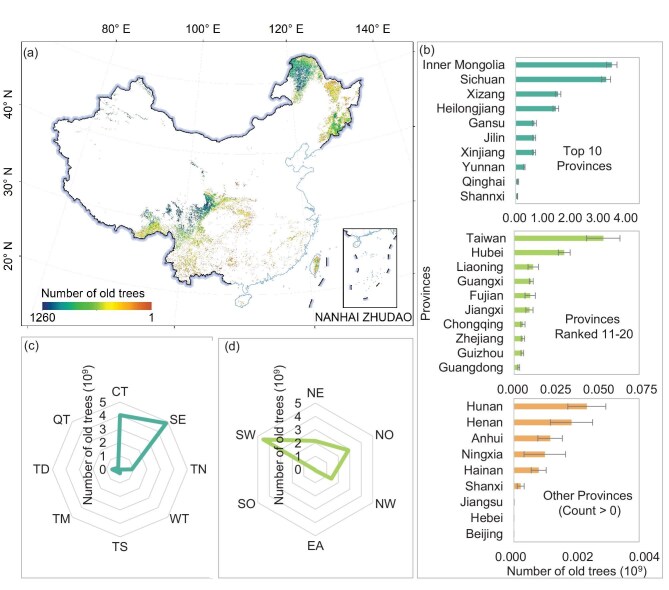
(a) Spatial distribution of old tree numbers across China. (b) The number of old trees for each province. (c) Statistical results of old tree numbers for each vegetation region. CT, cold temperate needleleaf forest; SE, subtropical evergreen broadleaf forest; TN, temperate needleleaf–broadleaf mixed forest; WT, warm temperate deciduous-broadleaf forest; TS, temperate steppe; TM, tropical monsoon forest-rainforest; TD, temperate desert; and QT, Qinghai–Tibet Plateau alpine vegetation. (d) The number of old trees for each geographical region. EA, east; SO, south; NW, northwest; SW, southwest; NE, northeast; and NO, north. The standard deviation of the estimated old tree number is illustrated by the error bars in (b). Review drawing number: GS 京(2026) 0643号.

Our analysis estimates 11.06 ± 0.30 billion old trees, with an average density of 48 ± 1 trees/ha in China’s forests. However, the national old tree density map (Fig. 1a) highlights the extreme spatial heterogeneity beneath this average value, underscoring the need for multi-scale analysis. Our analysis further indicates that the old-tree groups in China cover a total area of 42.77 ± 0.89 million hectares, accounting for 19% of the total forest area (Fig. S2). These groups are critical hotspots that harbour an estimated 10.86 ± 0.30 billion old trees, representing 98.19% of all old trees

identified. This pivotal result indicates that old trees in China’s forests exist predominantly within these concentrated communities rather than as isolated individuals, making the understanding and conservation of these groups paramount.

The distribution of old trees and old-tree groups is highly uneven across multiple scales (Tables S3 and S4). Among vegetation regions (Fig. 1c), the subtropical evergreen broadleaf forest region is the paramount

reservoir, maintaining not only the largest population (4.84 ± 0.20 billion trees, 43.78%) but also the largest old-tree group area (16.23 ± 0.48 million ha), accounting for 37.95% of the national total. However, this region exhibits a much lower old tree density (35 ± 1 trees/ha) due to its vast geographical extent. The cold temperate needleleaf forest region harbours the second-highest number of old trees (4.02 ± 0.13 billion, 36.40%) and the second-largest old-tree group area (13.68 ± 0.31 million ha). In contrast, compared with the subtropical region, it manifests a higher density (185 ± 3 trees/ha). The remaining vegetation regions collectively account for only 19.82% of China’s old trees. Notably, the temperate needleleaf–broadleaf forest region maintains the third-largest area of old-tree groups (7.66 ± 0.35 million ha), harbouring 0.88 ± 0.06 billion trees with a mean density of 33 ± 1 trees/ha. Furthermore, although the Qinghai–Tibet Plateau alpine vegetation and temperate desert regions contain relatively few old trees (0.18 ± 0.03 and 0.57 ± 0.05 billion, respectively), they exhibit extremely high old tree densities (548 ± 90 and 255 ± 16 trees/ha). In contrast, the warm temperate deciduous broadleaf forest (0.005 ± 0.002 billion), temperate steppe (0.30 ± 0.03 billion), and tropical monsoon forest regions (0.25 ± 0.02 billion) represent the minimal contributors to the national total.

Translating this ecological pattern into geographical regions reveals further insights (Fig. 1d). Southwest China has emerged as the primary reservoir of old trees, harbouring 4.52 ± 0.18 billion trees (40.89% of the national total) and maintaining the largest area of old-tree groups (14.86 ± 0.42 million ha). As a biodiversity hotspot, the complex terrain, such as the Hengduan Mountains, provides a crucial refugium [7,8]. Following the southwest, the north and northeast China serve as the secondary core regions, collectively accounting for 45.43% of the national total (2.88 ± 0.17 billion old trees in the north and 2.14 ± 0.12 billion old trees in the northeast). Notably, the north region exhibits the highest density nationwide (104 ± 3 trees/ha), while northeast retains the second largest extent of old tree groups (13.32 ± 0.51). This is attributable to their extensive natural forests, including the Greater and Lesser Xing’an Ranges [6]. In northwest China, a population of 1.39 ± 0.09 billion old trees is preserved with a second-highest density of 73 ± 2 trees/ha. Similar to previous observations, these forests are primarily confined to remote ranges such as the Qinling, Qilian, and Tianshan, which act as ‘ecological islands’ preserving high concentrations of old trees in an otherwise arid landscape [9–11]. In stark contrast, the east (0.08 ± 0.01 billion) and south (0.04 ± 0.004 billion) regions exhibit markedly low populations, low densities, and minimal areas of old-tree groups. This pattern is likely the legacy of intensive human activity and rapid urbanization within these densely populated zones, compounded by a higher frequency of natural disturbances.

At the provincial level (Fig. 1b), Inner Mongolia hosts the largest population of old trees in China (3.11 ± 0.17 billion). Other provinces exceeding the 1 billion thresholds include Sichuan (2.93 ± 0.15 billion), Xizang (1.37 ± 0.09 billion), and Heilongjiang (1.30 ± 0.09 billion). Collectively, the old tree groups of these four provinces account for 78.74% of the national total. Substantial populations exceeding 0.1 billion are found in Gansu, Jilin, Xinjiang, and Yunnan, while several other regions, including Qinghai, Shaanxi, Taiwan, Hubei, and Liaoning, maintain numbers exceeding 10 million. In terms of spatial extent, Inner Mongolia possesses the largest old-tree group (9.71 ± 0.45 million ha), followed by Heilongjiang (7.88 ± 0.38 million ha). Additionally, old-tree groups in Sichuan, Xizang, Jilin, Yunnan, Gansu, and Xinjiang all exceed the 1 million ha threshold. Notably, the area of old-tree groups within these eight provinces collectively accounts for 94.67% of the national total. Regarding old tree density, Xinjiang and Qinghai exhibit the highest densities nationwide (233 ± 13 and 220 ± 42 trees/ha, respectively), while Inner Mongolia, Sichuan, and Xizang also maintain high densities exceeding 100 trees/ha. Furthermore, mean densities exceeding 10 trees/ha are recorded in Gansu, Jilin, Heilongjiang, Taiwan, Ningxia, and Yunnan. This pronounced disparity in different provinces requires tailoring future conservation efforts to provincial realities through a differentiated investment of resources.

The conservation status of China’s old trees in forests was evaluated based on their distribution within the natural reserve network. In total, only 2.15 ± 0.12 billion old trees (19.43%) are located within formally national reserves. National-level reserves provide the principal safeguard (1.28 ± 0.10 billion trees), followed by provincial- (0.61 ± 0.06 billion), county- (0.15 ± 0.04 billion), and city-level (0.11 ± 0.03 billion) natural reserves (Table S3). While the density of old trees within natural reserves (66 ± 4 trees/ha) is higher than the national average (48 ± 1 trees/ha), it is notably lower than the densities observed in six individual provinces. Furthermore, only 19.75% of the total old-tree group is located within these reserves (8.45 ± 0.63 million ha), suggesting that many old tree groups remain unprotected. This gap arises because existing natural reserves were established with diverse objectives—such as safeguarding specific fauna, flora, or unique ecosystems—and were not designed specifically to protect these old trees. Consequently, conservation measures should also be implemented for old-tree groups outside formal reserves, particularly to maintain their integrity, which is vital for biodiversity conservation and carbon sequestration.

In summary, this study presents China’s first high-resolution distribution map of old tree density, concurrently revealing the national-scale distribution of old-tree groups. These findings establish a foundational digital archive and spatial map for old trees within China’s forests, bridging a critical knowledge gap that previously existed outside human-dominated landscapes and filling a significant data void for old tree conservation. This provides referable data to support the third national survey of old-tree groups. Furthermore, the map enables the identification of distribution hotspots, offering a scientific basis for targeted conservation and the establishment of conservation priority zones. Future research will focus on investigating the growth vigour and health status of the old-tree groups identified in this study, providing further scientific support for their active conservation.

## Supplementary Material

nwag197_Supplemental_File
