# Supplementary material for: China’s forests host a vast, previously unquantified population of old trees
Source: Natl Sci Rev. 2026 Apr 11;13(9):nwag197. doi: 10.1093/nsr/nwag197 (PMC13182250; doi:10.1093/nsr/nwag197)
Supplement: nwag197_Supplemental_File [file nwag197_supplemental_file.docx]

**Supplementary materials and methods**

**1 Methods**

To quantify the distribution and abundance of old trees ($age\geq100 years$) across China’s forest ecosystems, this study established a probabilistic inference framework based on structural moment inversion. By integrating multi-source remote sensing data to characterize forest vertical structural heterogeneity, the framework applies the theory of statistical moments to invert the distribution parameters of the population age structure at the pixel level, thereby achieving precise estimates of old tree density and spatial distribution.

**1.1 Data collection and preprocessing**

We employed four nationwide datasets to estimate the abundance and distribution of old trees in China, specifically: (1) the map of tree density in China at 100-m spatial resolution for 2020 (Cheng et al., 2025); (2) the map of forest age distribution in China at 30-m resolution for 2020 (Cheng et al., 2024); and (3–4) the maps of forest arithmetic mean height and weighted mean height in China at 30-m resolution for 2020 (Chen et al., 2024).

To unify the spatial scale for analysis, we resampled the 30-m resolution spatial data to 100-m resolution using a pixel aggregation and averaging method, thereby aligning them with the tree density map. The resulting input dataset comprised the total number of trees and key structural statistics for each 100-m grid cell.

**1.2 Theoretical Basis: The Weibull Demographic Model**

We selected the two-parameter Weibull distribution as a general probabilistic model to characterize the tree age structure within forest pixels. Owing to its flexibility, this distribution is widely employed in life cycle and survival analyses (Bailey & Dell, 1973; Diamantopoulou et al., 2015). Its probability density function is defined as:

|  | $f\left( t;k;\lambda\right)=\frac{k}{\lambda}{(\frac{t}{\lambda})}^{k-1}e^{{-(t/\lambda)}^{k}} (t\geq0)$ | (1) |
| --- | --- | --- |

Here, $t$ denotes tree age. The shape parameter $k$ determines the degree of heterogeneity in the population structure, while the scale parameter $\lambda$ reflects the characteristic life span of the population. Ecologically, $k$ serves as a distinct indicator: when $k\leq1$, the distribution exhibits a reverse J-shape, indicating uneven-aged forests characterized by abundant young trees and a long tail of old trees; conversely, when $k\geq1,$the distribution appears unimodal or normal-like, indicative of even-aged forests with a homogeneous structure.

**1.3 Parameter Retrieval via Structural Moment Inversion**

In this study, the arithmetic mean height ($H_{a}$) and the weighted mean height ($H_{w}$) are critical for quantifying intra-stand structure. $H_{a}$ represents the simple arithmetic mean of all tree heights within a stand, treating every individual with equal weight (Chen et al., 2024). In contrast, $H_{w}$ is the mean height weighted by individual tree basal area; since large trees dominate basal area, $H_{w}$ effectively captures the height characteristics of dominant canopy trees (Masaka et al., 2013).

Consequently, we constructed a structural heterogeneity index ($HI$) based on the ratio of $H_{w}$ to $H_{a}$ to reflect the degree of tree height irregularity within the stand. For instance, in even-aged forests or plantations, where tree sizes are uniform, these two metrics are nearly identical, resulting in a ratio approximating 1. Conversely, in uneven-aged forests, vertical stratification and gap regeneration lead to substantial size differentiation among individuals, causing the ratio to be significantly greater than 1. Thus, the $HI$ quantifies the skewness and dispersion of the within-stand height distribution, providing a robust physical basis for inverting the distribution parameters that characterize population structural complexity.

Building on this rationale, we estimated the Weibull parameters characterizing the within-stand age distribution based on the following hypothesis: at the pixel scale, the irregularity of the forest vertical structure (specifically, the shape of the tree height distribution) serves as a physical proxy for the complexity of the population age structure. Accordingly, leveraging our previously generated data products of $H_{a}$ and $H_{w}$, we applied the theory of statistical moments to estimate the Weibull parameters at the pixel level.

Specifically, according to the theory of statistical moments, the *n-th* raw moment (or moment about the origin) of a Weibull-distributed population is given by:

|  | $M_{n}=E\left[ X^{n} \right]=\text{λ}^{n}\text{Γ}\text{(1+}\frac{n}{k})$ | (2) |
| --- | --- | --- |

The $H_{a}$ corresponds to the first raw moment:

|  | $H_{a}\text{∝}M_{1}=\text{λ}^{1}\text{Γ}\text{(1+}\frac{1}{k})$ | (3) |
| --- | --- | --- |

The fundamental formula for the $H_{w}$ is given by:

|  | $H_{w}=\frac{\sum h_{i}\cdot g_{i}}{\sum g_{i}}$ | (4) |
| --- | --- | --- |

where $h_{i}$ denotes the height of the $i$-th tree, and $g_{i}$ represents the basal area of the $i$-th tree. The calculation for $g_{i}$ is given below, which is directly proportional to the square of the diameter at breast height ($D$):

|  | $g_{i}=\frac{\pi}{4}D_{i}^{2}$ | (5) |
| --- | --- | --- |

During the generation of our previous weighted mean height data, due to the unavailability of DBH information, we utilized the square of tree height ($h^{2}$) as the weighting factor. We demonstrated that this approach exhibits a significant correlation with traditional basal area weighting (Chen et al., 2024). Consequently, the calculation formula for the $H_{w}$ can be rewritten as follows:

|  | $H_{w}=\frac{\sum h_{i}\cdot h_{i}^{2}}{\sum h_{i}^{2}}=\frac{\sum H^{3}}{\sum H^{2}}=\frac{M_{3}}{M_{2}}$ | (6) |
| --- | --- | --- |

Combining the equations above, the ratio $HI$ can be expressed as follows:

|  | $HI=\frac{H_{w}}{H_{a}}=\frac{\text{Γ}(1+3/k)}{\text{Γ}(1+2/k)\text{Γ}(1+1/k)}$ | (7) |
| --- | --- | --- |

Consequently, $HI$ becomes a function dependent solely on the shape parameter $k$.

**1.4 Numerical Simulation and Model Parameterization**

Given that the relationship between $HI$ and $k$ involves complex ratios of Gamma functions and that the bias correction for weighted age lacks an analytical solution, we employed Monte Carlo numerical simulation to develop a parameter inversion model. Specifically, we constructed a synthetic dataset comprising 10,000 virtual forest stands. This dataset covers a broad spectrum of structural scenarios, ranging from extreme uneven-aged forests to highly homogeneous plantations.

**1.4.1 Simulation Experiment Design**

The simulation experiment was designed to derive the inversion function by utilizing generated paired data of "true values" and "observations". The specific steps are outlined as follows:

1. Parameter Space Sampling:

For each virtual forest stand, we randomly sampled the $k$ from the interval [0.5, 20]. This range fully encompasses the spectrum of forest stand types found in nature (where $k\leq1$ represents a reverse J-shaped distribution; $k\approx3.6$ approximates a normal distribution; and $k>10$ indicates a highly peaked distribution).

1. Generation of Virtual Plots

For each specified $(k,\lambda)$ combination, we employed the inverse transform sampling method based on the Weibull distribution to generate a collection of tree age samples comprising 10,000 virtual trees: $\{t_{1}, t_{2},\ldots,t_{n}\}$

(3) Calculation of Observational Metrics

Based on allometric scaling laws (assuming that basal area is proportional to the square of age), we calculated the simulated observational values for each virtual forest stand. These metrics included the $H_{a}$, $H_{w}$, weighted mean age (${Age}_{w}$), and the $HI$.

**1.4.2 Derivation of the Shape Parameter Retrieval Model**

Analysis of the simulated dataset revealed a strictly monotonically decreasing relationship between $HI$ and $k$ (Supplementary Figure 1). Specifically, as $k\to\infty$ (indicating extreme homogeneity), $HI\to1$; conversely, as $k\to0$ (indicating extreme heterogeneity), $HI$ exhibits a sharp increase.

To capture this non-linear characteristic, we employed the Levenberg-Marquardt algorithm to fit the following Power-law Decay Model:

|  | $k=\alpha\cdot{(HI-1)}^{-\beta}$ | (8) |
| --- | --- | --- |

Regression analysis demonstrated that the model exhibits an exceptionally high goodness of fit ($R^{2}>0.999, RMSE<0.05$). The derived parameters were $\alpha=1.405, \beta=0.545$. Consequently, this formula enables us to directly utilize our generated tree height products to precisely invert the morphology of the intra-stand age structure.

**1.4.3 Derivation of the Scale Parameter Bias Correction Model**

The simulation experiments further revealed a systematic bias between the ${Age}_{w}$ and the $\lambda$. Accordingly, we defined the bias ratio as:

|  | $\psi={Age}_{w}/\lambda$ | (9) |
| --- | --- | --- |

The results indicated that in uneven-aged forests, the $\psi$ value is substantially elevated. For instance, when $k=1$, $\psi=3$, implying that the observed weighted age is three times the true characteristic age. Conversely, in even-aged forests, $\psi=3$ approaches 1. To eliminate this bias induced by the weighting of large trees, we fitted a bias correction model (Supplementary Figure 2):

|  | $\psi\left( k \right)=1+\gamma\cdot k^{-\delta}$ | (10) |
| --- | --- | --- |

The fitting results are presented in Supplementary Figure 2, with the estimated parameters $\gamma=2.027$ and $\delta=3.340$. This model constitutes the core mechanism in our study for preventing the overestimation of old tree abundance, as it effectively restores the observed weighted age to the unbiased distribution parameter.

**1.5 China Old Tree Mapping**

Building upon the theoretical framework and models established above, we mapped the density and distribution of old trees across China at the national scale, following the steps outlined below:

(1) Calculation of $HI$ and $k$ Parameter Inversion

First, based on the maps of arithmetic mean height and weighted mean height across China, we calculated the $HI$ for each pixel. Subsequently, by applying Equation 8, we derived the pixel-level Weibull shape parameter ($k_{pixel}$).

(2) Reconstruction of $\gamma$ Parameter

We applied Equation 10 to calculate the scale correction factor $\psi\left( k_{pixel} \right)$. This factor was subsequently used to "de-bias" the input weighted mean age, thereby restoring the true scale parameter $\lambda_{pixel}$.

(3) Probability Integration and Density Estimation

Based on the derived parameter pair $(k_{pixel},\lambda_{pixel})$, we calculated the survival probability of old trees ($P_{old}$) within each pixel by integrating the probability density function over the interval $[100,\infty)$:

|  | $P_{old}=\int_{100}^{\infty} f(t;k_{pixel},\lambda_{pixel})dt=exp[-{(\frac{100}{\lambda_{pixel}})}^{k_{pixel}}]$ | (11) |
| --- | --- | --- |

Finally, the density of old trees within the pixel ($D_{old}$) was calculated as:

|  | $D_{old}=D_{total}\times P_{old}$ | (12) |
| --- | --- | --- |

Through a unified probabilistic inference framework, this method simultaneously resolves the challenge of identifying sparse, "long-tail" old trees in natural forests and detecting the overall distributional shift of old tree communities in mature plantations. Consequently, it achieves an adaptive and unbiased estimation across the diverse and complex forest ecosystems of China.

**1.6 Validation**

To assess the overall performance and reliability of the model, we collected a ground-truth validation dataset that was independent of the model construction process. This dataset records field survey information, enabling the verification of old tree classification (i.e., whether a tree qualifies as an old tree). We employed Overall Accuracy (*OA*) as the evaluation metric, calculated as shown in Equation 13.

|  | $OA= \frac{{correct\_num}_{ancient}}{{total\_num}_{ancient}}$ | (13) |
| --- | --- | --- |

where ${correct\_num}_{ancient}$ denotes the number of old trees correctly identified by this study within the validation dataset; ${total\_num}_{ancient}$ represents the total number of samples in the validation dataset.

**
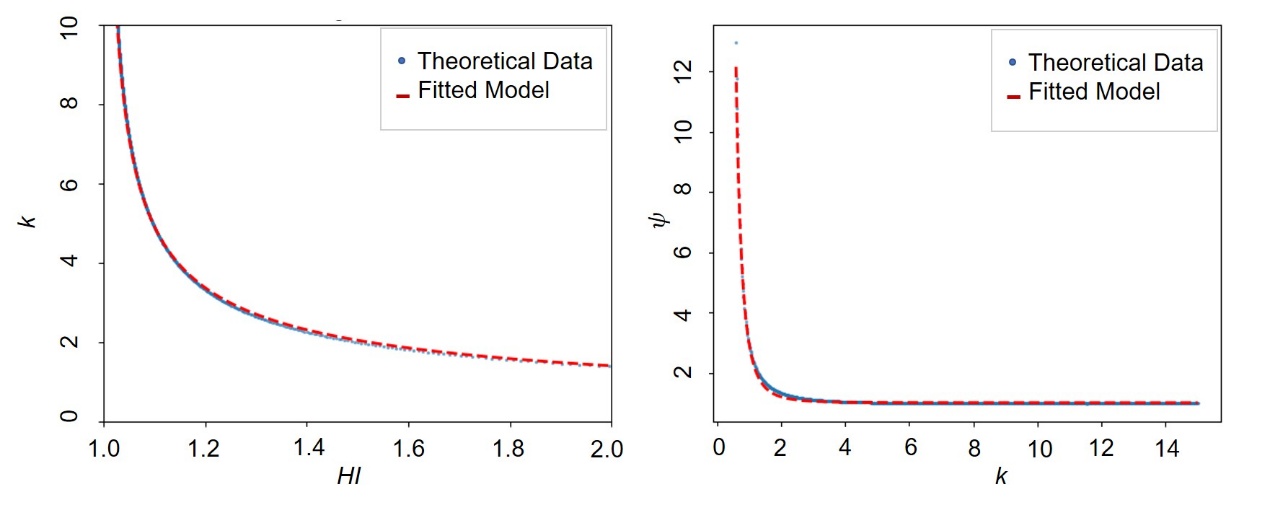
**

**Figure S1.**Calibration of the structure-dependent parameter retrieval models based on Monte Carlo simulations. (a) The non-linear inversion relationship between the *HI* and the Weibull shape parameter (*k*). As the ratio increases (indicating higher heterogeneity), k decreases following a power-law decay, reflecting an uneven-aged structure. (b) The relationship between the shape parameter (*k*) and the scale parameter bias correction factor ($\psi$). As k decreases, the correction factor increases significantly, helping remove the overestimation bias inherent in the weighted mean age.

**
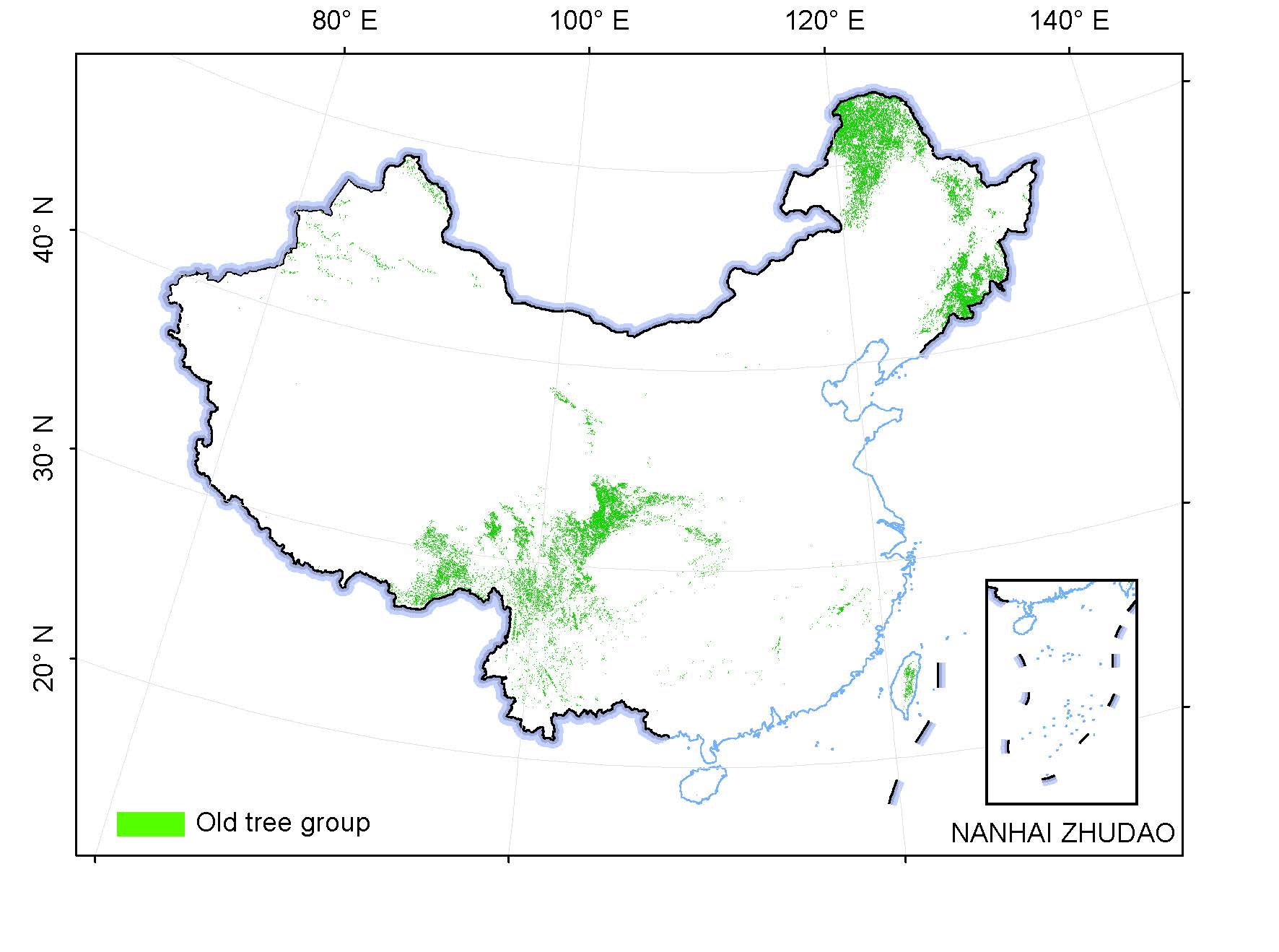
**

**Figure S2.** The national distribution of old-tree groups in China

**Table S1.** Descriptions of multiple remote sensing data used in the analysis of old trees

| Data type | Resolution | | Data source | Time |
| --- | --- | --- | --- | --- |
| Forest region | | 30 m | Planted and natural forest map (Cheng et al., 2023) | 2020 |
| Forest age | | 30 m | Forest age map (Cheng et al., 2024) | 2020 |
| Forest density | | 100 m | Forest density map (Cheng et al., 2025) | 2020 |
| Arithmetic mean height | | 30m | Forest arithmetic mean height | 2020 |
| Weighted mean height | | 30m | Forest weighted mean height | 2020 |
|  | |  |  |  |
| Vegetation region | | / | (Chinese Academy of Sciences, 2020) | 2020 |
| Provincial administrative divisions | | / | (National Geomatics Center of China, 2020) | 2020 |
| Nature reserve | | / | (China Nature Reserve Specimen Resource Sharing Platform, 2024) | 2012 |

**Table S2.** Summary of field validation sites and sample sizes used in this study

| Validation Site | Longitude | Latitude | Number of individual old tree plots | Number of old-tree group plots | Survey time |
| --- | --- | --- | --- | --- | --- |
| Hangzhou, Zhejiang | 120.17 | 30.28 | 25 | 3 | 2025 |
| Xianggelila, Yunnan | 99.85 | 27.94 | 1408 | 1408 | 2025 |
| Bome, Tibet | 95.77 | 30.05 | 211 | 211 | 2025 |
| Zayu, Tibet | 97.46 | 28.65 | 639 | 552 | 2022 |

*Note: The table summarizes the location, survey time, and sample size of the validation plots. The validation dataset is composed of two plot types based on their validation purpose: (1) "Individual Old Tree Plots" were survey plots designed to confirm the presence of at least one old tree; (2) "Old-Tree Group Plots" were survey plots designed to confirm if an area met the criteria to be classified as an old-tree group.*

**Table S3.** Old Tree number and density across multiple scales.

| Region | Region in detail | Total | CI | Density | CI |
| --- | --- | --- | --- | --- | --- |
| Vegetation | CT | 4023803119 | 137778987 | 185 | 2 |
| division | TN | 875064419 | 117068724 | 33 | 1 |
|  | TS | 297669746 | 68346830 | 44 | 2 |
|  | TD | 572500387 | 19515517 | 254 | 15 |
|  | WT | 5679261 | 68242298 | 1 | - |
|  | QT | 182672301 | 6940261 | 548 | 89 |
|  | SE | 4840141371 | 139195782 | 35 | 1 |
|  | TM | 257815514 | 25280822 | 17 | 1 |
| Geographical  region | Northeast | 2143966279 | 115536611 | 55 | 1 |
|  | South | 42860077 | 3781332 | 1 | - |
|  | East | 76451487 | 11488061 | 2 | - |
|  | North | 2878320574 | 172271476 | 104 | 2 |
|  | Northwest | 1393385067 | 89995049 | 72 | 2 |
|  | Southwest | 4520362634 | 182289063 | 72 | 1 |
| Province | Anhui | 1086606 | 367668 | 1 | - |
|  | Beijing | 606 | 430 | - | - |
|  | Chongqing | 5081584 | 1212294 | 1 | - |
|  | Fujian | 9297506 | 3033527 | 1 | - |
|  | Gansu | 603199135 | 65141048 | 96 | 4 |
|  | Guangdong | 2618387 | 519438 | - | - |
|  | Guangxi | 9900641 | 1115790 | 1 | - |
|  | Guizhou | 4730973 | 738754 | 1 | - |
|  | Hainan | 745312 | 224622 | - | - |
|  | Hebei | 2652 | 1041 | - | - |
|  | Henan | 1726340 | 631565 | - | - |
|  | Heilongjiang | 1298667697 | 90090110 | 56 | 1 |
|  | Hubei | 29093987 | 3452473 | 3 | - |
|  | Hunan | 2185073 | 569304 | - | - |
|  | Jilin | 598936920 | 47350442 | 63 | 2 |
|  | Jiangsu | 6058 | 2441 | - | - |
|  | Jiangxi | 8877722 | 2102820 | 1 | - |
|  | Liaoning | 11068778 | 3084164 | 2 | - |
|  | Inner Mongolia | 3109900105 | 168785592 | 168 | 5 |
|  | Ningxia | 926241 | 620918 | 16 | 6 |
|  | Qinghai | 74097424 | 23989795 | 220 | 42 |
|  | Shandong | 0 | 0 | - | - |
|  | Shanxi | 206488 | 104550 | - | - |
|  | Shaanxi | 55967701 | 7079964 | 6 | - |
|  | Shanghai | 0 | 0 | - | - |
|  | Sichuan | 2926263287 | 146084364 | 151 | 3 |
|  | Taiwan | 51855496 | 9715427 | 23 | 1 |
|  | Tianjin | 0 | 0 | 0 | 0 |
|  | Xizang | 1370554536 | 87326019 | 128 | 4 |
|  | Xinjiang | 593275810 | 45667989 | 232 | 13 |
|  | Yunnan | 279997369 | 20126602 | 13 | - |
|  | Zhejiang | 5075684 | 1048697 | 1 | - |
| Nature  Reserve | National-level | 1281942156 | 95080452 | 56 | 1 |
|  | Provincial-level | 610496280 | 62426088 | 89 | 3 |
|  | City-level | 107777434 | 26654792 | 114 | 11 |
|  | County-level | 148370911 | 35887388 | 74 | 5 |

**Note:** CT: Cold temperate needleleaf forest, SE: subtropical evergreen broadleaf forest, TN: temperate needleleaf-broadleaf mixed forest, WT: warm temperate deciduous-broadleaf forest, TS: temperate steppe, TM: tropical monsoon forest-rainforest, TD: temperate desert, QT: Qinghai-Tibet Plateau alpine vegetation

**Table S4.** Old-tree group area across multiple scales.

| Region | Region in detail | Old-tree group area (Million ha) | CI  (Million ha) |
| --- | --- | --- | --- |
| Vegetation division | Cold temperate needleleaf forest | 13.6789 | 0.3050 |
|  | Temperate needleleaf-broadleaf mixed forest | 7.6607 | 0.3545 |
|  | Temperate steppe | 1.4495 | 0.1274 |
|  | Temperate desert | 1.0755 | 0.0874 |
|  | Warm temperate deciduous-broadleaf forest | 0.0636 | 0.0259 |
|  | Qinghai-Tibet Plateau alpine vegetation | 0.2724 | 0.0490 |
|  | Subtropical evergreen broadleaf forest, | 16.2327 | 0.4776 |
|  | Tropical monsoon forest-rainforest | 2.3382 | 0.1697 |
| Geographical region | Northeast | 13.3239 | 0.5066 |
|  | South | 0.4461 | 0.0560 |
|  | East | 0.7122 | 0.0907 |
|  | North | 8.9733 | 0.4665 |
|  | Northwest | 4.452 | 0.2410 |
|  | Southwest | 14.864 | 0.4212 |
| Province | Anhui | 0.0101 | 0.0044 |
|  | Beijing | 0 | 0 |
|  | Chongqing | 0.0594 | 0.0181 |
|  | Fujian | 0.1077 | 0.0364 |
|  | Gansu | 2.2043 | 0.1865 |
|  | Guangdong | 0.0150 | 0.0064 |
|  | Guangxi | 0.0518 | 0.0109 |
|  | Guizhou | 0.0340 | 0.0080 |
|  | Hainan | 0.0029 | 0.0013 |
|  | Hebei | 0 | 0 |
|  | Henan | 0.0153 | 0.0083 |
|  | Heilongjiang | 7.8806 | 0.3794 |
|  | Hubei | 0.3699 | 0.0537 |
|  | Hunan | 0.0216 | 0.0079 |
|  | Jilin | 4.5591 | 0.2634 |
|  | Jiangsu | 0 | 0 |
|  | Jiangxi | 0.1094 | 0.0276 |
|  | Liaoning | 0.1337 | 0.0382 |
|  | Inner Mongolia | 9.7131 | 0.4500 |
|  | Ningxia | 0.0082 | 0.0050 |
|  | Qinghai | 0.1375 | 0.0400 |
|  | Shandong | 0 | 0 |
|  | Shanxi | 0.0021 | 0.0014 |
|  | Shaanxi | 0.7166 | 0.0885 |
|  | Shanghai | 0 | 0 |
|  | Sichuan | 7.7371 | 0.2966 |
|  | Taiwan | 0.4256 | 0.0662 |
|  | Tianjin | 0 | 0 |
|  | Xizang | 4.9010 | 0.2159 |
|  | Xinjiang | 1.2162 | 0.0860 |
|  | Yunnan | 2.2810 | 0.1303 |
|  | Zhejiang | 0.0585 | 0.0147 |
| Nature Reserve | National-level | 5.4671 | 0.2934 |
|  | Provincial-level | 2.2245 | 0.1899 |
|  | City-level | 0.3177 | 0.0634 |
|  | County-level | 0.4363 | 0.0866 |

**References**

1.Bailey, R., & Dell, T. R. (1973). Quantifying Diameter Distributions with the Weibull Function. *Forest Science*, *19*, 97-104.

2.Cheng, K., Chen, Y., Xiang, T., Yang, H., Liu, W., Ren, Y., Guan, H., Hu, T., Ma, Q., & Guo, Q. (2024). A 2020 forest age map for China with 30 m resolution. *Earth Syst. Sci. Data*, *16*(2), 803-819. <https://doi.org/10.5194/essd-16-803-2024>

3.Cheng, K., Su, Y., Guan, H., Tao, S., Ren, Y., Hu, T., Ma, K., Tang, Y., & Guo, Q. (2023). Mapping China’s planted forests using high resolution imagery and massive amounts of crowdsourced samples. *ISPRS Journal of Photogrammetry and Remote Sensing*, *196*, 356-371. <https://doi.org/https://doi.org/10.1016/j.isprsjprs.2023.01.005>

4.Cheng, K., Yang, H., Chen, Y., Yang, Z., Ren, Y., Zhang, Y., Lin, D., Liu, W., Huang, G., Xu, J., Chen, M., Qi, Z., Xu, G., Tao, S., Guan, H., Ma, Q., Wan, H., Hu, T., Su, Y., . . . Guo, Q. (2025). How many trees are there in China? *Science Bulletin*, *70*(7), 1076-1079. <https://doi.org/https://doi.org/10.1016/j.scib.2025.02.001>

5.Chen, Y., Yang, H., Yang, Z., Yang, Q., Liu, W., Huang, G., Ren, Y., Cheng, K., Xiang, T., Chen, M., Lin, D., Qi, Z., Xu, J., Zhang, Y., Xu, G., and Guo, Q.(2024). Enhancing high-resolution forest stand mean height mapping in China through an individual tree-based approach with close-range lidar data. *Earth System Science Data. 16*, 5267–5285. https://doi.org/10.5194/essd-16-5267-2024

6.China Nature Reserve Specimen Resource Sharing Platform. (2024). List and Vector Boundaries of Nature Reserves in China. *Zenodo*. https://doi.org/10.5281/zenodo.14875797

7.Chinese Academy of Sciences (2020). Vegetation region of China. *Resource and Environment Science and Data Platform*. https://www.resdc.cn/data.aspx?DATAID=133

8.Diamantopoulou, M. J., Özçelik, R., Crecente-Campo, F., & Eler, Ü. (2015). Estimation of Weibull function parameters for modelling tree diameter distribution using least squares and artificial neural networks methods. *Biosystems Engineering*, *133*, 33-45. <https://doi.org/https://doi.org/10.1016/j.biosystemseng.2015.02.013>

9.Masaka, K., Sato, H., Torita, H., Kon, H., and Fukuchi, M.(2013). Thinning effect on height and radial growth of Pinus thunbergii Parlat. trees with special reference to trunk slenderness in a matured coastal forest in Hokkaido, *Japan. Journal of Forest Research, 18*, 475–481. https://doi.org/10.1007/s10310-012-0373-y.

10.National Geomatics Center of China. (2020). Provincial administrative divisions of China. *National Platform for Common Geospatial Information Services*. https://example.com/dataset-link

11.Su, Y., Guo, Q., Hu, T., Guan, H., Jin, S., An, S., Chen, X., Guo, K., Hao, Z., Hu, Y., Huang, Y., Jiang, M., Li, J., Li, Z., Li, X., Li, X., Liang, C., Liu, R., Liu, Q., . . . Ma, K. (2020). An updated Vegetation Map of China (1:1000000). *Science Bulletin*, *65*(13), 1125-1136. <https://doi.org/https://doi.org/10.1016/j.scib.2020.04.004>

12.Teimouri, M., Hoseini, S. M., & Nadarajah, S. (2013). Comparison of estimation methods for the Weibull distribution. *Statistics*, *47*(1), 93-109. <https://doi.org/10.1080/02331888.2011.559657>
